# Touchscreen and Translational Cognition: A Systematic Review of Trials in Humans and Rodents

**DOI:** 10.1111/jnc.70297

**Published:** 2025-11-14

**Authors:** Tamires Coelho Martins, Renata Maria Silva Santos, Rayany Karolyny da Silva Andrade, André Soares da Silva, Felipe Baptista Brunheroto, Isabella Paula Gomes Rocha, Vitória Carrazza Gambogi Loureiro, Yuri Cristelli de Sousa Silva, Ana Caroline Nogueira Souza, Eduardo de Souza Nicolau, Débora Marques Miranda, Marco Aurélio Romano‐Silva

**Affiliations:** ^1^ Center for Technology in Molecular Medicine (CTMM), Federal University of Minas Gerais (UFMG) Belo Horizonte Minas Gerais Brazil; ^2^ Post‐graduate Program in Molecular Medicine Federal University of Minas Gerais (UFMG) Belo Horizonte Minas Gerais Brazil; ^3^ Department of Pediatrics Federal University of Minas Gerais (UFMG) Belo Horizonte Minas Gerais Brazil; ^4^ Department of Psychiatry Federal University of Minas Gerais (UFMG) Belo Horizonte Minas Gerais Brazil

**Keywords:** co‐clinical, cognition, human, rodent, systematic review, touchscreen platform

## Abstract

The implementation of touchscreen platforms in co‐clinical trials for rodents (i.e., mice and rats) and humans to assess cognitive functions presents an opportunity to overcome barriers present in conventional clinical trials. To better visualize the progress made in this area, this review proposes a systematic synthesis of the comparability of touchscreen cognitive assessment studies applied to both humans and rodents in a co‐clinical framework. To accomplish this objective the Ovid, PubMed, Scopus and ScienceDirect databases were searched, in English, and without publication date limit and registered on the International Prospective Register of Systematic Review (PROSPERO) under the number CRD420250650537. The screening resulted in 5 cross‐sectional studies and 1 randomized controlled trial (RCT) included, which were assessed for methodological quality and risk of bias using the Joanna Briggs Institute (JBI) critical appraisal tools. The data acquired in this review reinforce the potential of touchscreen platforms for cognitive assessment across human and rodent models. Behavioral flexibility and visuospatial cognition excelled in terms of comparability. The scarcity of studies and methodological diversity represent significant gaps in the field. Regardless, the available data highlight important opportunities for advancing translational research in cognition with a co‐clinical approach.

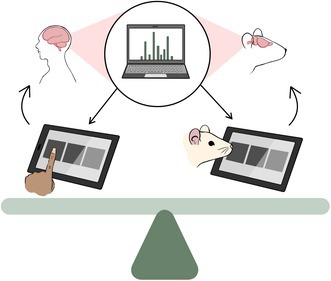

## Graphical abstract

The implementation of touchscreen platforms in co‐clinical trials for rodents (i.e., mice and rats) and humans to assess cognitive functions presents an opportunity to overcome barriers present in conventional clinical trials. A systematic review was conducted to assess the comparability of the use of touchscreen cognitive assessment in a co‐clinical framework addressing humans and rodents. The data acquired reinforce the potential of touchscreen platforms for cognitive assessment. Behavioral flexibility and visuospatial cognition excelled in terms of comparability. The available data highlight important opportunities for advancing translational research in cognition with a co‐clinical approach.

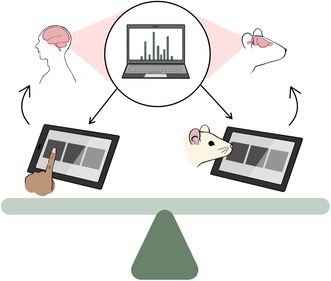

**Abbreviations**
5C‐CPT: 5‐choice continuous performance test
APL: acute promyelocytic leukemia
BF: Bayes Factor
CANTAB: Cambridge Neuropsychological Test Automated Battery
CIRP: Co‐Clinical Imaging Research Resource Program
D‐amp: dextroamphetamine
DLG2: discs large homolog 2
EMOTICOM: emotional and social function battery
HD: Huntington's disease
IED: intra‐extra dimensional set‐shifting
JBI: Joanna Briggs Institute
KO: knock‐out
MoCA: Montreal Cognitive Assessment
NCI: National Cancer Institute
OCD: obsessive‐compulsive disorder
PAL: paired‐associate learning
PECO: Population, Exposure, Comparator, and Outcomes
PR: progressive ratio
PRISMA: Preferred Reporting Items for Systematic Reviews and Meta‐Analyses
PROSPERO: Prospective Register of Systematic Review
RCT: randomized controlled trial
RVP: rapid visual information processing
SWM: spatial working memory
TUNL: trial‐unique nonmatching to location
WT: wildtype

## 1 Introduction

Co‐clinical trials have emerged as an innovative approach by enabling real‐time data integration between experiments conducted in humans and animal models, promoting greater accuracy and relevance in the results obtained (Nardella et al. 2011). This study modality aims to solve the delays present in the usual preclinical‐clinical model and was initially used in the search for a cure for acute promyelocytic leukemia (APL), with successful results, as a “preclinical‐clinical” approach (Nardella et al. 2011; Balasubramanian et al. 2021). Although it is a field of current interest, a notable peak in publications and citations in this field occurred between 2013 and 2015, coinciding with increased support from the National Cancer Institute (NCI) for precision medicine, and its Co‐Clinical Imaging Research Resource Program (CIRP) (Dumont et al. 2021; Zhang 2023). However, there is a lack of systematic literature reviews on co‐clinical studies to support decision‐making and support researchers in the neuroscience field.

The distribution of resources for conducting co‐clinical research is not homogeneous around the world; therefore rodents (i.e., mice and rats) are generally preferred when conducting animal research (Carr 2025). Although nonhuman primates share remarkable similarities to humans due to shared evolutionary history, their use in research has been dwindling because of maintenance costs, difficult access to specialized labor, several ethical reasons, and the “3 Rs” of research (Replacement, Reduction, and Refinement), aimed at replacing animals with alternative models whenever possible, reducing the number of animals used, and refining procedures in order to minimize suffering (Harding 2017; Lopresti‐Goodman and Villatoro‐Sorto 2022). Rodents, however, can be cheaper to maintain and can be housed in smaller facilities; they are extensively used due to their physiological and genetic similarities to humans, as well as their shorter life cycle, which allows for faster observation of biological effects and more reliable anticipation of expected clinical results in humans (Carr 2025; Soufizadeh et al. 2024).

Conventional studies have traditionally been conducted in a fragmented manner, with isolated preclinical and clinical trials. This leads to methodological discrepancies between experiments, incomparable results, and challenges to develop new therapies (Salmon et al. 2024). This translational disconnection is a major barrier in the development of therapies targeting neuropsychiatric conditions (Salmon et al. 2024). Despite numerous studies aimed at the development of new treatments, many drugs have failed in clinical trials (Reuben et al. 2024; Granzotto et al. 2024). In neuroscience, one of the reasons for this high failure rate is the difficulty in having animal models that faithfully represent human psychiatric symptoms, both from a biological perspective and in the behavioral tasks used for assessment (Lee et al. 2021).

There is a discrepancy in the application of cognitive tests in rodents and those used in humans. While rodents are often evaluated using mazes, humans are assessed using broader tools such as the computerized tasks from the Cambridge Neuropsychological Test Automated Battery (CANTAB) or paper‐and‐pencil tests, which cover multiple domains of cognition (Lee et al. 2021). Recent advances, such as automated touchscreen platforms free from experimenter interference, have reduced stress and variability, allowing for more accurate and reproducible behavioral analyses (Huang et al. 2023; Horner et al. 2013). Touchscreen‐based tests emerge as a tool that offers greater precision and standardization, allowing humans and rodents to respond to visual stimuli in a similar manner (Bussey et al. 2012; Horner et al. 2013). While human participants touch the screen with their finger, rodents interact with the stimulus by touching with their snout (Palmer et al. 2021). The adaptation of CANTAB‐inspired paradigms to rodents increased the translational validity of the tests, enabling direct comparisons with human assessments (Palmer et al. 2021). Paired‐Associates Learning (PAL), for example, assesses associative memory and, in aged rats, revealed changes in connectivity between the hippocampus, prefrontal cortex, and the retrosplenial region, suggesting preservation of neural plasticity (Gaynor et al. 2023). Tasks such as PAL and Trial‐Unique Nonmatching to Location (TUNL), when applied intensively, also improved cellular plasticity and cognitive performance in Alzheimer's models, resulting in increased synaptogenesis and improved cognitive phenotype (Shepherd et al. 2021). These tests have also been useful for identifying deficits after central nervous system injuries, such as trauma or stroke, with high accuracy in assessing memory, cognitive flexibility, and learning (Cotter et al. 2023). The integration of touchscreen technology with features such as electroencephalograms and optogenetics has enabled the simultaneous recording of behavior and neural activity, with high temporal resolution and minimal manual interference. This has enabled detailed correlations between neurophysiological variables and behavioral measures such as reaction time, number of errors, and exploration patterns (Kangas et al. 2021; Piantadosi et al. 2025).

The benefits of using touchscreen technology in rodents have facilitated the investigation of specific cognitive processes by neuroscientists from different specialties, using high standardization, minimal experimenter interference, and strong translational potential (Horner et al. 2013). The translational value stems from the similarity between the tasks employed in both rodents and humans, which confers a degree of face validity. Face validity refers to how much a task appears to measure the same psychological construct across species, based on similarities in procedures and structure. This differs from construct validity, which assesses whether the task truly measures the intended cognitive process, and from predictive validity, which examines whether task performance can forecast real‐world or clinically relevant outcomes. While face validity alone is not sufficient to guarantee these other forms of validity, it increases the likelihood of achieving them when tasks are designed to mimic human paradigms closely (Bussey et al. 2012). These advantages have contributed to the growing popularity and relevance of touchscreen‐based assessments in modern neuroscience (Dumont et al. 2021).

Because they generate highly consistent data over time, touchscreen platforms are especially suitable for integrating advanced experimental methodologies, benefiting both basic neuroscience research and drug discovery efforts. Furthermore, their potential for integration with state‐of‐the‐art neural recording technologies makes these platforms particularly effective in multiplexed studies (Carr 2025). However, for co‐clinical trials with touchscreen devices to be successful, standardization is necessary. To this end, the community must synchronize its efforts, and inspiration can be drawn from the success of this approach in the field of oncology. Seeking to integrate preclinical and clinical research, we observed the need to understand the possibilities of using touchscreens, given their sensitivity to detect cognitive deficits in animal models, especially in rodents. Considering the reality of population aging and the increase in neurodegenerative and neuropsychiatric diseases, the demand for viable techniques for conducting simultaneous co‐clinical studies in this area became evident. These studies provide, in addition to diagnostic accuracy, practical applicability and ethical obviousness in the study of cognition. In addition, co‐clinical studies that applied touchscreens can show ways to maximize the use of touchscreens in translational protocols and strengthen the scientific basis that supports their application in clinical practice. For this reason, we conducted a systematic review with the objective of identifying primary studies that reported synchronous co‐clinical investigations using touchscreens for cognitive assessment in humans and rodents.

## 2 Method

A systematic review was conducted adhering to the Preferred Reporting Items for Systematic Reviews and Meta‐Analyses (PRISMA) (Page et al. 2021) and registered in the International Prospective Register of Systematic Reviews (PROSPERO), under the number CRD420250650537, based on the guiding question “how comparable are the results for cognitive tests using touchscreen platforms in neuropsychiatric disorders in a co‐clinical approach for humans and rodents?”. The population, exposure, comparator, and outcomes (PECO) strategy was adopted, and the population consisted of humans and mice or rats, exposed to the touchscreen platform, compared to their respective control groups. The outcome expected was the comparability in cognitive function evaluation.

From the guiding question the following descriptors were used: (touchscreen operant system) AND (rat) OR (mice) AND (translational) OR (translational science) OR (translational research) AND (humans) AND (cognitive test) AND (cognition) in all fields on the Ovid, PubMed and Scopus databases, and in titles, keywords and abstract on ScienceDirect, in English and without publication date limit. Citation search in gray literature was not performed.

The inclusion criteria were experimental articles that investigate cognitive processes in mice/rats and humans using touchscreen technology, written in English. The exclusion criteria set were nonexperimental studies and preprints, studies in other languages aside from English, studies that investigated only rats/mice or only humans, studies that used a platform other than touchscreen for the mice/rats, studies with any other animal that are not mice/rats, and studies with nonhuman primates. The decision to exclude nonhuman primates was based on careful methodological and practical consideration. Rodent models are well established and widely validated in the scientific literature, serving as a robust reference for preclinical studies. Evidence consistently shows that rats and mice produce results that are highly compatible with human findings across multiple fields, including pharmacology, behavioral neuroscience, and studies of neuropsychiatric disorders. Given this strong translational potential, we focused on assessing whether such compatibility also holds in the emerging context of touchscreen‐based cognitive testing.

### 2.1 Screening Procedure

**FIGURE 1:**
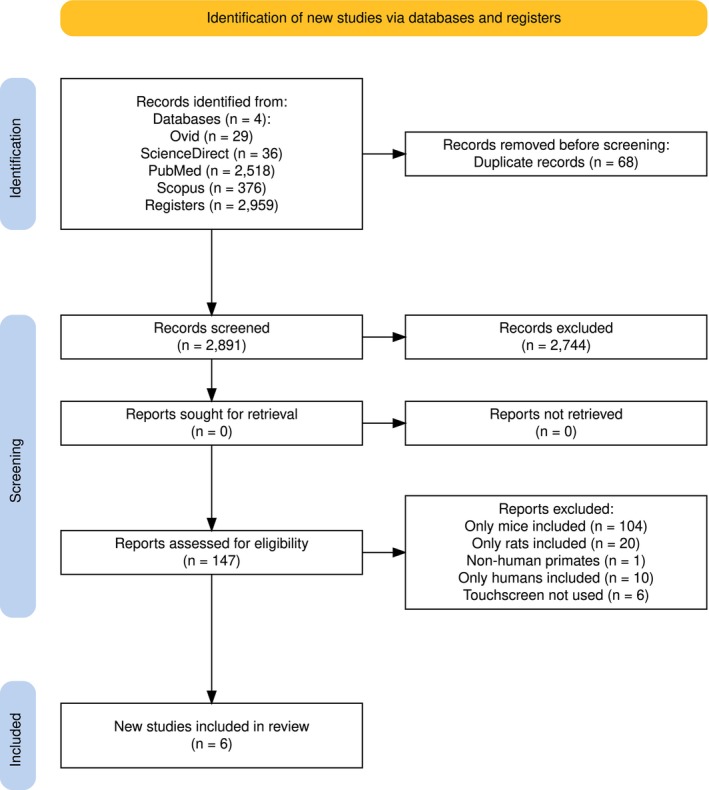
PRISMA fluxogram.

The screening was conducted through peer review using the Rayyan (Ouzzani et al. 2016) platform to organize, manage, and screen the articles. Initially, duplicate articles were eliminated as described in Figure 1 (PRISMA fluxogram). Conflicts between the reviewers regarding eligibility were discussed and resolved by consensus with a third member. Afterwards, the selected articles were read in full, and only those that effectively met the established criteria were included.

### 2.2 Data Extraction

Data were extracted using a standardized spreadsheet that included the following data: first author, year of publication, country, study type, article objective, sample characteristics, such as age, sex, and species, type of touchscreen intervention including the name of the test battery and the specific task, and outcome measures. The data extraction process was conducted in pairs.

### 2.3 Quality Assessment

The methodological quality and risk of bias of the included studies were assessed using the “Critical Appraisal Tools” developed by the Joanna Briggs Institute (JBI) for cross‐sectional (Aromataris et al. 2024) and randomized controlled trials (RCTs) (Barker et al. 2023). Rather than relying on a numerical scoring system, this tool provides conceptual guidance to support the reviewer's judgment. Based on this qualitative assessment, studies were categorized as having good, moderate, or poor methodological quality, depending on the presence and severity of design or implementation flaws.

Using a critical and qualitative approach, cross‐sectional studies were rated as high quality when all appraisal criteria were met; as moderate quality when items related to confounding factors (Q5 and Q6) were inadequately addressed; and as low quality when other key methodological elements (Q1, Q4, and Q8) were missing (see Table 3). Regarding RCTs, studies were considered high quality when all criteria were fulfilled; moderate quality when concerns were raised about randomization (Q1, Q6, and Q13) or allocation procedures (Q2); and low quality when other critical methodological domains (Q3, Q4, Q5, Q7, Q9, Q10, and Q11) were not adequately addressed (see Table 4). The complete table with the questions included, is available as Data S1.

## 3 Results

See Figure 1.

### 3.1 Global Distribution

The six selected articles were published between 2012 and 2021, comprising four cross‐sectional studies and one randomized clinical trial. Collectively, they include 372 human participants and an estimated sample of 293–353 mice. The global distribution is presented in Table 1.

**TABLE 1:** Distribution by country.

Country	Number of studies	Human subjects	Mice sample
Australia	1	140	45
France	1	80	52
UK	3	148	96–106^a^
USA	1	4	100–150^b^
Total	6	372	~293–353

*Note:* ~ Estimated value based on previous data from the table.

^a^ Estimated value based on the methodology described by Nithianantharajah et al. (2015).

^b^ Estimated value based on the methodology described by Nithianantharajah et al. (2013).

### 3.2 Study Characteristics

Most studies (66.7%) used transgenic or knockout mice, them being *Sapap3* knock‐out mutant mice (*Sapap3* KO) (Benzina et al. 2021), R6/1 (Heath et al. 2019) and Dlg1^+/−^, Dlg2^−/−^, Dlg3^−/y^, and Dlg4^−/−^ (Nithianantharajah et al. 2013, 2015). Only two studies did not use wildtype (WT) mice as controls, also being the only studies to use C57BL/6 mice (MacQueen et al. 2018), and one of them had the mice modeled for photothrombotic stroke (Chow et al. 2020). Studies predominantly (83.4%) evaluated a condition, either genetic (Nithianantharajah et al. 2013, 2015) or health‐related (Benzina et al. 2021; Heath et al. 2019; Chow et al. 2020). A single study had its main goal on the outcome after pharmacological intervention (MacQueen et al. 2018).

### 3.3 Participants and Animal Subjects' Characteristics

Participants' ages ranged from 20 to 81 years (weighted mean 60.8 pooled standard deviation 13.5) and animal subjects' ages ranged from 1.4 to 7 months (weighted mean 3.3 pooled standard deviation 0.2). In the pooled participant sample, there was a nearly equal participation of males (45.9%), though one study did not provide complete gender data for all human samples (Benzina et al. 2021). In the pooled animal sample, only male subjects were included. Data from the included studies are detailed in Table 2.

**TABLE 2:** Characteristics of the studies included in the review.

Author (year), country, study type	Objective	Sample, intervention	Findings
Benzina et al. (2021), France, cross‐sectional	To investigate behavioral flexibility in compulsive behaviors in obsessive‐compulsive disorder (OCD) patients and *Sapap3* KO mice	**Humans, *N*: 80** *N*: 40 control/OCD Mean age (SD): 40.28 (13.59) control, 40.15 (13.22) OCD Gender: 15 males control/OCD Intervention: Reversal learning task **Mice, *N*: 52** *N*: 26 WT/*Sapap3* KO Age in days (SD): 200.12 (12.23) WT, 199.35 (12.29) *Sapap3* KO Gender: all males Intervention: Reversal learning task	OCD or *Sapap3* KO gene did not have an impact on performance (Bayes Factor (BF)_Inclusion_ < 1 for group factor). There was not a difference in the number of trials to reach the reversal criterion (BF_10_ = 0.64, *d* = 0.27 [0.04 0.77]), nor mice (BF_10_ = 0.7, *d* = 0.33 [−0.1 0.92]). There was no group difference in the number of reversal errors (humans: BF_10_ = 1.5, *d* = 0.35 [0.07 0.88]; mice: BF_10_ = 0.44, *d* = 0.25 [−0.2 0.83]). OCD “checkers” displayed more behavioral inflexibility (BF_+0_ = 4.66, d = 0.74 [0.08 1.25]). Impaired *Sapap3* KO presented more behavioral inflexibility than unimpaired *Sapap3* KO mice and WT controls (BF_10_ > 100, *d* = 1.37 [0.54 2.17]). There were differences of spontaneous strategy change probability between the three subgroups (humans: BF_10_ = 1.19, *η* ^2^ = 0.08; mice: BF_10_ > 100, *η* ^2^ = 0.41).
Chow et al. (2020), Australia, cross‐sectional	To evaluate poststroke cognitive function in human chronic stroke survivors and photothrombotic stroke mice model	**Humans, *N*: 140** *N*: 70 stroke/nonstroke Mean age (SD): 61.9 (13.8) stroke, 64.6 (10.0) nonstroke Gender: 38 males stroke, 24 males nonstroke Intervention: CANTAB, PAL task **Mice, *N*: 45** *N*: 20 sham, 24 stroke Age: 7–8 weeks Gender: all males Intervention: PAL task	Stroke survivors made fewer correct responses for the first time, in comparison to participants within the control group (*p* = 0.002). Mice modeled for stroke had worse performance in comparison to the sham group (*p* = 0.032). There was a significant inverse association between stroke and the first attempt memory score was present in humans (crude *β* (95% CI), −2.26 (−3.62 to −0.899), *p* = 0.001), as for mice there was a significant effect of stroke (*F*(1, 18) = 5.65; *p* = 0.029) and time (*F*(6, 108) = 15.1; *p* < 0.0001) on the mean correct rate. Stroke survivors had a higher number of attempts to successfully complete an attempt (*p* = 0.015). Mice modeled for stroke needed longer times to complete a session (*p* < 0.0001) and completed a reduced number of tasks (*p* < 0.0001).
Heath et al. (2019), England, cross‐sectional	In this present study they aimed to demonstrate the validity of using touchscreen‐delivered progressive ratio tasks to mirror apathy assessment in Huntington's disease (HD) patients and a representative mouse mode	**Humans, *N*: 43** *N*: 23 HD, 20 control Mean age (SD): 53.6 (14.6) HD, 52.2 (20.2) control Gender: 13 HD males, 10 control males Intervention: Emotional and social function battery (EMOTICOM) **Mice, *N*: 52** *N*: 23 R6/1, 29 WT Age: 6 weeks Gender: all males Intervention: Progressive ratio	Motivation was higher for controls in both species (humans: *p* < 0.001, *d* = 1.6; mice: *p* < 0.001; *d* = 1.09), supported by post reinforcement pause (humans: *p* < 0.001, *d* = 1.4; mice: *p* < 0.001; *d* = −1.53). There was a significant reduction in R6/1 animals relative to WT littermates, corresponding to a lower intrinsic motivational baseline (*p* < 0.001; *d* = 1.96). For humans, the highest number of touchscreen responses in the patient group was highly correlated to functional decline in everyday activities and lower scores indicated worse impairment (*r* = 0.45, *p* < 0.05).
MacQueen et al. (2018), England, RCT	To investigate how D‐amp would improve 5C‐CPT performance in a similar manner for both healthy adult mice and human participants, demonstrating pharmacological validity for the task	**Humans, *N*: 71** Age: 18–35 years Gender: 47.9% male Intervention: 5‐choice continuous performance test (5C‐CPT) **Mice, *N*: 32** Age: ~8 weeks at onset of testing Gender: all males Intervention: 5C‐CPT	For humans, D‐amp significantly improved performance at both the 10 and 20 mg doses, relative to placebo (*d* = 0.821 and 0.758; *p* < 0.05), such a result was also seen at the 0.3 mg/kg in mice relative to saline (*d* = 1.138). The effect was driven by increased hit rate in both species (humans: *p* = 0.001, *d* = 0.938 and 0.903; mice: *p* = 0.017, *d* = 1.197) and concurrent reduced percent omissions in humans (*p* = 0.001; *d* = 0.897 and 0.807) at both doses, and for mice at the smaller dose, although it did not reach statistical significance (*p* = 0.055). The number of correct responses was also improved in the two species, by both doses of D‐amp for humans (*p* < 0.001; *d* = 1.115 and 1.076) and at the 0.3 mg/kg dose of D‐amp relative to saline for mice (*p* = 0.002; *d* = 1.676). In mice improved accuracy only happened at the 0.3 mg/kg dose, while the 1.0 mg/kg reduced accuracy (*d* = 0.845). Hit reaction‐time variability was, however, reduced by both doses (*p* < 0.001; *d* = 1.001 and 1.338) for humans, and for mice it had no significant effect (*p* = 0.859).
Nithianantharajah et al. (2013), USA, cross‐sectional	To examine the genetic basis of the vertebrate cognitive repertoire through paralogous genes and compare homologous cognitive processes in mice and humans	**Humans, *N*: 4** Age: 24–67 years old Gender: 1 male Intervention: Spatial working memory (SWM), intra‐extra dimensional set‐shifting (IED), PAL, Rapid Visual Information Processing (RVP) **Mice, *N*: ~10–15 for each cohort** *N*: Dlg1^+/−^, Dlg2^−/−^, Dlg3^−/y^, Dlg4^−/−^ and WT Age: undisclosed Gender: all males Intervention: Pavlovian conditioned approach, visual discrimination and reversal learning task, object‐location paired‐associates learning task, extinction, five‐choice serial reaction time task (5‐CSRTT)	Mice with Dlg2^−/−^ and humans with mutations in discs large homolog 2 (DLG2) made significantly more errors than healthy control subjects from the general population in tests of visual discrimination acquisition and cognitive flexibility (*p* < 0.005) and visuo‐spatial learning and memory (*p* < 0.005). Humans with mutations in DLG2 also showed decreased accuracy compared to controls in a test for sustained attention (*p* < 0.005), similar to the impaired response accuracy seen in Dlg2^−/−^ mice.
Nithianantharajah et al. (2015), England, cross‐sectional	To assess mice and humans carrying disease‐related genetic mutations with an identical touchscreen based cognitive test	**Humans, *N*: 34** *N*: 4 DLG2, 30 controls Age: 23–67 years old Gender: all females DLG2, 14 male controls Intervention: Adapted rodent touchscreen object‐location paired associates learning test **Mice, *N*: ~10–15 per group** *N*: ~10–15 Dlg2^−/−^/WT Age: undisclosed Gender: all males Intervention: Rodent touchscreen object‐location paired associates learning test	Dlg2^−/−^ mice presented a striking impairment in object‐location paired associates learning, performing consistently on an average of 50% chance level on all three blocks of trials (*p* < 0.01). Human controls displayed progressive acquisition of object‐location paired associates across training trials (*p* < 0.001), akin to WT mice, without gender or IQ making a difference on performance (*p* = 0.74). Individuals with DLG2 CNV deletions tested on the same test failed to show this progressive acquisition (*p* = 0.549), still performing approximately at chance level.

### 3.4 Quality Assessment

See Tables 3 and 4.

**TABLE 3:** Quality assessment for cross‐sectional studies.

Author (year)	Q1	Q2	Q3	Q4	Q5	Q6	Q7	Q8	Quality
Benzina et al. (2021)	★	★	★	★	★	★	★	★	High
Chow et al. (2020)	★	★	★	★	★	★	★	★	High
Heath et al. (2019)	★	★	★	★	★	☆	★	★	Moderate
Nithianantharajah et al. (2013)	★	★	★	★	☆	☆	★	★	Moderate
Nithianantharajah et al. (2015)	★	★	★	★	☆	☆	★	★	Moderate

*Note:* ★, yes; ☆, no.

**TABLE 4:** Quality assessment for randomized controlled trials.

Author (year)	Q1	Q2	Q3	Q4	Q5	Q6	Q7	Q8	Q9	Q10	Q11	Q12	Q13	Quality
MacQueen et al. (2018)	★	★	★	★	★	☆	★	★	★	★	★	★	☆	Moderate

*Note:* ★, yes; ☆, no.

### 3.5 Findings

In Benzina et al. (2021) results were similar between humans and mice, when comparing performance profiles in a reversal event in subjects with OCD and controls. Similar performance profiles could be observed after a reversal event between compulsive subjects and their controls in the two species, meaning OCD or the knockout of the *Sapap3* gene did not have an impact on performance (BF_Inclusion_ < 1 for group factor). The number of trials needed to reach reversal criterion did not differ between compulsive and control groups, neither for human subjects (BF_10_ = 0.64, *d* = 0.27 [0.04 0.77]), nor for mice (BF_10_ = 0.7, *d* = 0.33 [−0.1 0.92]). Similarly, no significant group differences were found in the number of reversal errors, neither in humans (BF_10_ = 1.5, *d* = 0.35 [0.07 0.88]), nor in mice (BF_10_ = 0.44, *d* = 0.25 [−0.2 0.83]). In OCD patients, correlation analysis showed that disease severity and task performance were not related. Likewise, there was no correlation in mice between grooming level and the main behavioral parameters. A post hoc analysis revealed that OCD “checkers” needed more trials than both OCD “noncheckers” (BF_+0_ = 4.66, *d* = 0.74 [0.08 1.25]) and healthy controls (BF_+0_ = 9.32, *d* = 0.67 [0.14 1.17]), therefore displaying more behavioral inflexibility. Similarly, “impaired” *Sapap3* KO presented more behavioral inflexibility than “unimpaired” *Sapap3* KO mice and WT controls (BF_10_ > 100, *d* = 1.37 [0.54 2.17]). There was a positive correlation between the severity of “checking” symptoms and the probability of spontaneous strategy change, that is, changing its response despite positive feedback. These results suggested that OCD “checkers” had a high response lability as identified through an elevated spontaneous strategy change probability. The subgroup analysis in both species supported this result, with differences in spontaneous strategy change probability observed between the three subgroups either in humans (BF_10_ = 1.19, *η* ^2^ = 0.08) or in mice (BF_10_ > 100, *η* ^2^ = 0.41).

Similarly, in the study by Chow et al. (2020), individuals affected by stroke demonstrated notably fewer correct responses when attempting to recall the appropriate patterns for the first time, in comparison to participants within the control group (*p* = 0.002). Likewise, mice that experienced a stroke displayed poorer performance, achieving a markedly lower accuracy rate in comparison to the sham group (*p* = 0.032). For humans, a significant inverse association between stroke and the first attempt memory score was present (crude *β* (95% CI), −2.26 (−3.62 to −0.899), *p* = 0.001), as for mice there was a significant effect of stroke (*F*(1, 18) = 5.65; *p* = 0.029) and time (*F*(6, 108) = 15.1; *p* < 0.0001) on the mean correct rate, meaning that stroke considerably affects the capacity to retain and manipulate visuospatial information, supported by the increased number of attempts to successfully complete each level by stroke survivors (*p* = 0.015), similarly observed by mice's longer times to complete a session (*p* < 0.0001), still completing a reduced number of tasks (*p* < 0.0001).

When investigating the use of touchscreen‐delivered progressive ratio tasks as an assessment tool for apathy in Huntington's disease, Heath et al. (2019) found that, assessed by breakpoint value, motivation was higher for controls in both species (humans: *U* = 394.0, *p* < 0.001, *d* = 1.6; mice: *t*(48.162) = 4.0879; *p* < 0.001; *d* = 1.09). Post reinforcement pause corroborated this finding (humans: *U* = 56.0, *p* < 0.001, *d* = 1.4; mice: *t*(29.606) = −5.0861; *p* < 0.001; *d* = −1.53). Once again, the comparison of the estimated peak progressive ratio response rate indicated a significant reduction in R6/1 animals relative to WT littermates, corresponding to a lower intrinsic motivational baseline (*p* < 0.001; *d* = 1.96). For humans, the highest number of touchscreen responses in the patient group was highly correlated to functional decline in everyday activities and lower scores indicated worse impairment (*r* = 0.45, *p* < 0.05).

MacQueen et al. (2018) investigated the effects of D‐amp on the 5C‐CPT in humans and mice. For humans, D‐amp significantly improved performance at both 10 and 20 mg doses, relative to placebo (*d* = 0.821 and 0.758; *p* < 0.05); such a result was also seen at 0.3 mg/kg in mice relative to saline (*d* = 1.138). The effect was driven by an increased hit rate in both species (humans: *F*(2, 136) = 7.628, *p* = 0.001, *d* = 0.938 and 0.903; mice: *F*(3, 28) = 4.015, *p* = 0.017, *d* = 1.197) and concurrent reduced percent omissions in humans (*F*(2, 68) = 7.350, *p* = 0.001; *d* = 0.897 and 0.807) at both doses, and for mice at the smaller dose, although it did not reach statistical significance (*F*(3, 28) = 2.852, *p* = 0.055). The number of correct responses was also improved in the two species, by both doses of D‐amp for humans (*F*(2, 136) = 12.037, *p* < 0.001; *d* = 1.115 and 1.076) and at the 0.3 mg/kg dose of D‐amp relative to saline for mice (F(3,28) = 6.012, *p* = 0.002, *d* = 1.676). Although D‐amp improved accuracy for humans at both doses, in mice it only happened at the 0.3 mg/kg dose, while the 1.0 mg/kg reduced accuracy (*d* = 0.845). Hit reaction‐time variability was, however, reduced by both doses (*F*(2, 68) = 12.964, *p* < 0.001; *d* = 1.001 and 1.338) for humans, and for mice it had no significant effect (*F*(3, 28) = 0.252, *p* = 0.859). For humans and mice, the false alarm rate, hit reaction‐time, and premature responses were not significantly impacted by D‐amp at any dose.

Nithianantharajah et al. (2013, 2015) describe the same experiment in two separate papers, where the 2013 paper focuses on mice and the 2015 paper focuses on humans. Nithianantharajah et al. (2013) investigated the performance of humans with DLG2 gene mutation and mice lacking the Dlg2 gene (Dlg2^−/−^) on a touchscreen‐based cognitive test. In agreement with results from Dlg2^−/−^ mice, humans with mutations in DLG2 made significantly more errors than healthy control subjects from the general population in tests of visual discrimination acquisition and cognitive flexibility (*p* < 0.005) and visuo‐spatial learning and memory (*p* < 0.005). In addition, humans with mutations in DLG2 also showed decreased accuracy compared to controls in a test for sustained attention (*p* < 0.005), an effect similar to the impaired response accuracy seen in Dlg2^−/−^ mice. When the standardized performance score was calculated, it exhibited a negative score for both Dlg2^−/−^ mice and DLG2 humans, representing a performance lower than average. In Nithianantharajah et al. (2015) the rodent touchscreen object‐location PAL test was adapted to humans, therefore they considered it an identical test. Results showed that, compared to the WT control mice, which showed a progressive increase in performance, Dlg2^−/−^ mice presented a striking impairment in object‐location paired associates learning, performing consistently on an average of 50% chance level on all three blocks of trials (*p* < 0.01). Human controls displayed progressive acquisition of object‐location paired associates across training trials (*p* < 0.001), akin to WT mice, without gender or IQ making a difference on performance (*p* = 0.74). And again, individuals with DLG2 CNV deletions tested on the same test failed to show this progressive acquisition (*p* = 0.549), still performing approximately at chance level.

## 4 Discussion

This systematic review compiled studies that necessarily investigated humans and rodents together, which, to our knowledge, had not been done before. Despite broadening the scope to include rats and mice, only co‐clinical studies with mice were identified. Overall, the studies analyzed in this systematic review consistently highlight the translational potential of touchscreen platforms for cognitive assessment across human and rodent models in conditions such as DLG2 CNV deletions, OCD, HD, stroke and pharmacological intervention. For the domains of learning and memory, motivation, attention and reaction time, the quality of evidence was lower, although still considerable. Comparability, however, was considered only when similar or equivalent experimental paradigms or interventions were implemented.

Benzina et al. (2021) reported parallel profiles of impaired behavioral flexibility in both humans diagnosed with obsessive‐compulsive disorder and their *Sapap3* KO mice counterparts. Both groups exhibited comparable deficits in reversal learning tasks and increased response lability, demonstrating the potential for the translational applicability of touchscreen cognitive tasks in examining OCD‐related behavioral phenotypes across species. A pivotal step in the success of the APL preclinical‐clinical approach was the stratification of patients and mice through genetic testing, allowing higher specificity for treatment (Piazza et al. 2001; He et al. 1997; Grisolano et al. 1997). Interestingly, subtle behaviors, such as “checking” and “not checking” in patients with OCD were stratified, with the same being then observed in mice as impaired and unimpaired (Benzina et al. 2021). It is important to note that excessive grooming has been used for years as a classical measure of compulsion in animal models of OCD, but it has not correlated with cognitive results obtained using touchscreens. Although grooming is a typical rodent behavior, it tends to intensify in situations of novelty or stress, which limits its specificity as a marker of compulsion (Zike et al. 2017). In contrast, touchscreen tasks aligned with human findings, suggesting that the animal model captures truly translational aspects when using this type of paradigm. Thus, the touchscreen‐derived data not only validate the model but also suggest that these measures may be more robust than classic behavioral markers (Kangas and Bergman 2017). These findings reinforce that the use of cognitive paradigms, such as touchscreens, strengthens the translational validity of animal models of OCD.

Similarly, the translational utility demonstrated by Chow et al. (2020) identifies touchscreen‐based tests of cognition as promising tools for measuring post‐stroke impairments in both rodent and human cognition. Importantly, the observed consistency in visuospatial impairments using the touchscreen‐based PAL task aligns well with evidence that stroke location and extension, as well as lesion characteristics, are major determinants of cognitive outcomes (Schellhorn et al. 2021; Weaver et al. 2021). Strokes affecting strategic areas like the damage of parietal lobes, frontal‐temporal cortices, or thalamic regions typically lead to strong impairment across executive functioning, attention, and memory (Weaver et al. 2021). However, Chow et al. (2020) notably relied on self‐reported stroke history, and the lack of detailed neuroanatomical characterization may limit interpretability regarding lesion‐specific cognitive deficits. Standard screening measures like the Montreal Cognitive Assessment (MoCA) (Nasreddine et al. 2005) and targeted neuropsychological testing remain widely used in post‐stroke evaluation and, unlike self‐reported stroke history, provide objective measurement of domain‐specific impairment (Kosgallana et al. 2019; Stolwyk et al. 2021), though their sensitivity and specificity vary across cognitive domains and cohorts, and they may still miss subtle deficits that could be captured by touchscreen paradigms in controlled experimental settings (Burton and Tyson 2015; Kosgallana et al. 2019). Therefore, touchscreen measures like PAL are very suitable as complementary tools because they can quantify domain‐specific cognition, diminish examiner bias with automation, and provide direct interspecies comparison by employing nearly identical versions in rodent and human models (Hvoslef‐Eide et al. 2015). Notably, touchscreen measures can identify subtle and specific impairments in cognition, especially in visuospatial learning, attention, and memory, and many of these paradigms already incorporate internal controls for motor and perceptual confounds. For instance, the CANTAB battery includes a Motor Screening Task (MOT) to assess basic visuomotor speed and accuracy before engaging in more demanding cognitive tasks and tests, allowing researchers to distinguish performance deficits caused by motor or sensory impairment from those due to cognitive dysfunction (Cambridge Cognition 2023). Furthermore, future translational research with touchscreen paradigms would benefit from incorporating detailed neuroimaging and lesion mapping to correlate cognitive outcomes more precisely with anatomical injury, enhancing interpretability and generalizability for interspecies comparisons following stroke.

Further supporting the translational potential, Heath et al. (2019) also provided evidence for the appropriateness of touchscreen‐based progressive ratio (PR) tasks for measuring motivational impairments in Huntington's disease. In their study, patients and R6/1 mice displayed significantly lower breakpoints and decreased running rates indicative of apathy‐like motivational impairments due to fronto‐striatal dopamine dysfunction characteristic of the disease (Martínez‐Horta et al. 2018). This cross‐species consistency attests to the PR task's translational robustness in measuring effort‐based motivational mechanisms and confirms its construct in HD research. Thus, the use of automated touchscreen tests favors standardization and interspecific comparability, strengthening its application in translational research on neuropsychiatric symptoms, such as apathy (Martínez‐Horta et al. 2018).

Furthermore, research on DLG2 gene mutations by Nithianantharajah et al. (2013, 2015) highlights touchscreen tests' translational power in measuring genetic disruption impacts on cognition. Individuals with heterozygous deletions in DLG2 and Dlg2 KO mice demonstrated congruent impairments in cognitive flexibility, attention, and visuospatial memory with very similar or identical touchscreen‐based cognition paradigms. These findings add to the construct validity and sensitivity of touchscreen tests, demonstrating that conserved disrupted cognition caused by DLG2 mutations is measurable similarly across species. Limited by small human sample size and differences in developmental compensation, these studies nonetheless clearly demonstrate the value of touchscreen paradigms for genetic studies on cognition, enabling direct translational comparisons and therapeutic investigation. Co‐clinical trials are also applicable to rare neurodevelopmental disorders associated with variants in the DLG2 gene, as illustrated in the studies analyzed, following a line inspired by strategies previously adopted in genetic syndromes such as APL. Due to systematic review method constraints, the articles Nithianantharajah et al. (2013, 2015) were considered two separate publications, as they were returned from the databases. However, they present results from the development of a single study. Although the general conclusion remains, this further limits the variety of outcomes present and reinforces methodological flaws related to vague descriptions, hindering the accuracy of the conclusions.

While most outcomes exhibited translational comparability, some remarkable differences at the species level were noted, above all in pharmacological scenarios. MacQueen et al. (2018) examined cognitive enhancements produced by D‐amp with the 5C‐CPT in both human subjects and mice. Both species exhibited improved attention (greater hit rate, accuracy, and signal detection) without impulsive escalations. However, species differences were evident in reaction time variability and dose–response effects, underlining intrinsic pharmacokinetic and neurobiological species differences. These results point to crucial aspects in translational pharmacology, with an emphasis on a need for precisely calibrated dose–response paradigms and species‐specific pharmacokinetic modeling to realistically interpret cross‐species cognitive measures. Considering co‐clinical trials were first intended as a platform to research the cure for APL (Balasubramanian et al. 2021; Nardella et al. 2011), there are still few studies directly comparing its potential in different areas, meaning it is yet to be demonstrated how comparable they will be on different pharmacological pathways. Additionally, interindividual variability presents another challenge for such trials, as they happen in both humans and mice, worsening reproducibility and amplifying the chances of data misinterpretation (Löscher 2024). Unfortunately, none of the included studies mentioned releasing their data in open science platforms or other communities, despite their existence, which hinders the dissemination of knowledge, and the desirable synchronization needed for co‐clinical trials to thrive (Dumont et al. 2021). The entire community would greatly benefit from the dissemination of knowledge on online communities such as the touchscreencognition.org.

It is essential to acknowledge that, although the same test was used in the study by MacQueen et al. (2018), the method of application differed between the two species. Humans used a joystick to select responses, while animals interacted with the test by poking the screen with their snout. This may introduce a bias in the study, as the use of a touchscreen requires less refined motor control (Daud et al. 2020). Therefore, this difference in the method of application can be seen as a factor contributing to variations in the results obtained in these studies.

Methodological quality varied among the included studies, which influences the degree of reliability of translational inferences (Barker et al. 2023). High methodological quality ratings were assigned to cross‐sectional studies assessing behavioral flexibility (Benzina et al. 2021) and visuospatial cognition (Chow et al. 2020). These studies met all criteria, particularly regarding clearly defined participant characteristics, validated cognitive tasks, and robust statistical analyses accounting for potential confounders. In contrast, moderate‐quality ratings were assigned to studies investigating motivation (Heath et al. 2019), cognitive flexibility, attention, and visuospatial memory (Nithianantharajah et al. 2013, 2015), primarily due to inadequate identification and management of confounding variables. Similarly, the RCT by MacQueen et al. (2018), assessing attention and reaction time, showed methodological rigor in several areas but was limited by the absence of outcome assessor blinding postintervention and unaddressed deviations from standard RCT designs, increasing detection bias risks. The variability in methodological rigor emphasizes the importance of domain‐specific considerations when interpreting translational evidence. Although most of the reviewed articles identified significant outcomes comparable in both humans and rodents, the studies' major limitations regard methodological standardization. It would have been relevant for the researchers to perform an age comparison between the animals and the humans investigated. All the animals used in the studies were young adult rodents (6–8 weeks), which is especially important in studies involving cohorts with very distinct age ranges, such as those in the 23–63 years (Nithianantharajah et al. 2015), 25–75 years (patients with HD), and 20–81 years (controls) (Heath et al. 2019). While the study by Heath et al. (2019) matched the ages of the human controls, the same attention was not given to the animals. Still, there is evidence that with aging, significant changes occur in cognition, such as sensory perception, processing speed, memory formation, and retention, which peak in early adulthood and tend to decline progressively with aging, affecting performance in various cognitive domains in a nearly linear fashion (Murman 2015; Brito et al. 2023). In tasks such as delay eyeblink conditioning, elderly humans show impairments, as do elderly rodents, whose performances are also impaired, as evidenced in a study that used the Barnes Maze to test spatial memory (Brito et al. 2023). A study conducted by Shoji et al. (2016) performed a large‐scale analysis of behavioral data obtained from a battery of tests with 1739 WT mice, aged between 2 and 12 months, and observed significant behavioral differences between age groups, with older groups showing reduced locomotor activity, changes in fear memory, and increased anxiety‐like behavior. Similarly, Shoji and Miyakawa (2019) found, in another study with different behavioral batteries, that older mice exhibit decreased locomotor activity, increased anxiety, worsened motor performance, and memory decline, as well as changes in prepulse inhibition and depression‐like behavior. Thus, age has a considerable impact on the assessment of cognition. Considering the use of animals of age comparable to human participants would make the research more robust and precise, contributing to a better translation of the results to the human context.

Additionally, all the studies used male mice. Though the studies by Nithianantharajah et al. (2015) and Chow et al. (2020) included both female patients and control groups, they exclusively used male mice. Similarly, the study by MacQueen et al. (2018), which has the highest prevalence of female patients (52.1%), also used only male mice. It should be noted that the studies included in this review did not find any relevant associations with gender or did not have a significant effect on the variables; yet, the literature indicates that the use of female mice remains restricted due to concerns about variation induced by the estrous cycle. However, in co‐clinical studies, it is ideal that the experimental conditions approximate those of the target population, and choosing males may limit translational validity, compromising the consistency of results and making it difficult to apply findings to women (Wiseman 2023). Alas, Levy et al. (2023) demonstrated that female behavior is not significantly affected by the estrous cycle, although females display more individualized behavioral patterns compared to males, as well as greater behavioral stability. In this context, it is important to consider that despite the genetic similarities between men and women, there are anatomical and brain connectivity differences: men tend to have, on average, a larger total brain volume, while women show greater connectivity in the default mode network (Arenaza‐Urquijo et al. 2024). Differences are also observed in cognitive domains, with men generally performing better in specific visuospatial tasks, while women excel in verbal tasks (Ramos‐Loyo et al. 2022). Furthermore, distinctions are found in the incidence of cognitive disorders: conditions such as Alzheimer's disease, major depressive disorder, as well as eating disorders and generalized anxiety disorder, are more prevalent in women, while schizophrenia, autism spectrum disorder, attention‐deficit/hyperactivity disorder, and conduct disorder are more commonly diagnosed in men (Lee et al. 2022; Hines 2020). A study by Cañete and Giménez‐Llort (2021) demonstrated sex‐specific differences in nociceptive biomarkers in Alzheimer's models, and Statsenko et al. (2022) observed distinct patterns and rates of brain atrophy between sexes in humans. These findings reinforce that biological sex has a significant influence on cognition and, although a stratified analysis by sex and age was not possible given the data available from the gathered studies, they must be considered in future studies to ensure greater accuracy in translational research.

This review highlighted the importance of synchronous co‐clinical trials, both to anticipate the outcomes of clinical studies in humans and to support the design and analysis of these studies (Chen et al. 2012). Despite technological advances and the growing interest in the application of co‐clinical paradigms, especially those involving touchscreen testing, the simultaneous conduct of experiments in humans and rodents still faces significant barriers. For these studies to fulfill their translational potential, it is essential that detailed operational information is readily accessible. The use of touchscreen technology, widely recognized by neuroscientists for closely mirroring tests applied in humans and animals, would also benefit from this accessibility. There are currently initiatives, such as virtual support platforms, with training and collaborative forums, that seek to facilitate their implementation (Dumont et al. 2021). However, what was observed was a scarcity of available co‐clinical studies, as well as frequent flaws in methodological reporting, which compromise critical evaluation and weakens the potential for rigorous replication of these experiments.

The development of co‐clinical trials requires standardization of protocols, expertise of the researchers involved, and rigor in scientific reporting. Even with certain weaknesses in the reporting of the experiments, it is now evident that the process of implementing the co‐clinical approach is now in the phase of identifying highly comparable rodent models, and the next step may be to test this comparability in a wide range of drugs as well. Based on what we observed in this review, future research initiatives in this area should consider age and sex as critical variables, and include neuroimaging and lesion mapping, to improve the correlation between cognitive deficits and anatomical damage, favoring more accurate interspecies comparisons.

## 5 Limitations

The absence of methodological standardization and insufficient detailing in the included articles represents a significant limitation. Standardized and comparable protocols may save money, decrease risks, and amplify the chances to develop new drugs. Given the scarcity of studies observing humans and rats or mice simultaneously it was not viable to focus on specificities, meaning the results should not be interpreted in such light. Additionally, the inclusion of two articles resulting from the development of a single study reduced the diversity of methodologies evaluated in this review. Another limitation regards the absence of more recent studies; since technology is rapidly evolving it is likely that the results are outdated for today's standards.

## 6 Conclusions

A step more was given in standardization and comparability of co‐clinical platforms for cognitive assessment in neuropsychiatric disorders. Despite the few studies, there is good quality of evidence showing how promising they will be for research. Future studies will benefit from addressing the problems hindering the success of the co‐clinical approach in the neuroscience field.

## Author Contributions

**Tamires Coelho Martins:** conceptualization, investigation, writing – original draft, methodology, data curation. **Renata Maria Silva Santos:** conceptualization, methodology, writing – review and editing, supervision. **Rayany Karolyny da Silva Andrade:** conceptualization, investigation, writing – original draft. **André Soares da Silva:** investigation, writing – original draft, project administration. **Felipe Baptista Brunheroto:** investigation, writing – original draft. **Isabella Paula Gomes Rocha:** conceptualization, investigation, writing – original draft. **Vitória Carrazza Gambogi Loureiro:** conceptualization, investigation. **Yuri Cristelli de Sousa Silva:** conceptualization, investigation. **Ana Caroline Nogueira Souza:** methodology. **Eduardo de Souza Nicolau:** methodology. **Débora Marques Miranda:** writing – review and editing, supervision. **Marco Aurélio Romano‐Silva:** funding acquisition, supervision.

## Conflicts of Interest

The authors declare no conflicts of interest.

## Peer Review

The peer review history for this article is available at https://www.webofscience.com/api/gateway/wos/peer‐review/10.1111/jnc.70297.

## Supporting information


**Data S1:** jnc70297‐sup‐0001‐supinfo.zip.

## Data Availability

All data supporting the findings of this study are available within the article.
